# Cardiotocography in breech versus vertex delivery: an examiner-blinded, cross-sectional nested case-control study

**DOI:** 10.1186/s12884-016-1115-5

**Published:** 2016-10-21

**Authors:** Elli Toivonen, Outi Palomäki, Heini Huhtala, Jukka Uotila

**Affiliations:** 1 School of Medicine, University of Tampere, 33014 Tampere, Finland; 2 Department of Obstetrics and Gynecology, Tampere University Hospital, PL 2000, 33521 Tampere, Finland; 3 School of Health Sciences, University of Tampere, 33014 Tampere, Finland

**Keywords:** Fetal monitoring, Breech presentation, Cardiotocography, Vaginal breech delivery

## Abstract

**Background:**

The safety of vaginal breech delivery has been debated for decades. Although it has been shown to predispose infants to immediate depression, several observational studies have also shown that attempting vaginal breech delivery does not increase perinatal morbidity or low Apgar score at the age of five minutes. Cardiotocography monitoring is recommended during vaginal breech delivery, but comparative data describing differences between cardiotocography tracings in breech and vertex deliveries is scarce. This study aims to evaluate differences in intrapartum cardiotocography tracings between breech and vertex deliveries in the final 60 min of delivery. A secondary goal is to identify risk factors for suboptimal neonatal outcome in the study population.

**Methods:**

One hundred eight breech and 108 vertex singleton, intended vaginal deliveries at term from a tertiary hospital with 5000 annual deliveries were included. Two experienced obstetricians, blinded to fetal presentation, neonatal outcome and actual mode of delivery, evaluated traces recorded 60 min before delivery. They provided a three-tier classification and evaluated different trace features according to FIGO (1987) guidelines. Factors associated with acidemia and low Apgar scores were identified by univariate and multivariable analyses performed with binary logistic regression. Student’s *T*-test and chi-square test were used, as appropriate.

**Results:**

Late decelerations were seen in 13.9 % of breech and 2.8 % of vertex deliveries (*p* = 0.003) and decreased variability in 26.9 % of breech and 8.3 % of vertex deliveries (*p* < 0.001). In multivariable analysis complicated variable decelerations and breech presentation were identified as risk factors for neonatal acidemia and low Apgar score at the age of five minutes. Pathological trace and breech presentation were independent risk factors for low Apgar score at the age of one minute.

**Conclusions:**

Decreased variability and late decelerations were more prevalent in breech compared to vertex deliveries. Pathological trace predicts immediate neonatal depression and especially complicated variable decelerations may signal more severe distress. Further research is needed to create guidelines for safe management of vaginal breech delivery.

## Background

Cardiotocography (CTG), the monitoring of fetal heart rate and uterine contractions, has been used in an attempt to assure fetal well-being during labor for over forty years [1]. Despite ongoing debate on the efficacy of the method [2–5], continuous CTG monitoring has become widely used and is recommended during labor of women with high-risk conditions [6, 7]. Several rating systems for normal and abnormal CTG tracings have been developed, of which the three-tier model, also presented in International Federation of Obstetrics and Gynaecology (FIGO) guidelines in 1987 [7], is probably the most widely accepted consensus yet reached [8, 9]. However, even when following a predefined rating system, inter- and intraobserver agreement has been described to vary [9–11].

Breech presentation occurs in 3–4 % of term, singleton deliveries [12]. The optimal mode of delivery remains controversial, as retrospective studies both encouraging [13–15] and discouraging [16] attempted vaginal delivery have been published. Three randomized controlled studies of the topic have been published with contrasting results [17–19], the latest and most extensive suggesting a policy of routine cesarean delivery [17]. In our hospital, a trial of vaginal delivery is allowed if strict selection criteria are met, and the threshold to convert the mode of delivery to an emergency cesarean section (CS) is kept low during labor. Continuous CTG monitoring is recommended throughout labor to detect fetal distress [20, 21]. Mothers giving birth to a breech infant should be thoroughly informed on the contrasting research data and the choice regarding the mode of delivery should be made in collaboration of the mother and the health care provider [20]. As many mothers will choose vaginal delivery, more studies on improving the safety of vaginal breech delivery is needed, as indicated in the latest Cochrane review on the mode of delivery in breech presentation [22].

Studies on breech deliveries dated as early as the 1970’s and 1980’s have described frequent decelerations during the first stage of labor [23, 24] and one study observing term breech fetuses in second stage of labor described a high prevalence of variable decelerations, thought to be produced by compression of the umbilical cord [25]. Furthermore, the non-stress antepartum fetal heart rate traces of breech fetuses have been described to show decreased variability compared to vertex fetuses [26], which in turn was associated with a shorter umbilical cord. A recent study demonstrated that fetal ST-waveform analysis was also applicable in breech vaginal deliveries, although intervention was more often triggered by a preterminal CTG than ST changes in breech deliveries compared to vertex deliveries [27]. However, based on a search of MEDLINE (English language; 1961 – October 2015; search terms “Breech presentation” and “Cardiotocography”), no comparative studies concerning the differences in intrapartum CTG traces between breech and vertex deliveries have been published. As several institutions around the world still allow trial of vaginal delivery also in breech presentation, evidence-based data on safe clinical management of vaginal breech delivery is indicated.

The objective of this study was to determine whether CTG tracings at the late phase of the first stage and during the second stage of labor differ between fetuses in breech and vertex presentation. A secondary aim was to identify risk factors for suboptimal neonatal outcome in the study population.

## Methods

The study protocol was approved by the Pirkanmaa Hospital District’s ethical committee (decision R12236). All intended singleton vaginal term breech deliveries, ending in either spontaneous vaginal delivery or emergency cesarean section, between January 2007 and April 2009 were included in the study if the quality of the CTG tracing was deemed adequate. The control group consisted of intended vaginal vertex deliveries, ending in either spontaneous vaginal, operative vaginal, or emergency CS delivery. The groups were matched by the actual mode of delivery, either spontaneous vaginal or operative delivery. Thus the number of spontaneous deliveries was equal in the groups, and the number of acute CS in the breech group was equal to the number of acute CS’s and vacuum extractions in the vertex group. The total number of deliveries in the study was 216 (108 deliveries in each group).

**Fig. 1:**
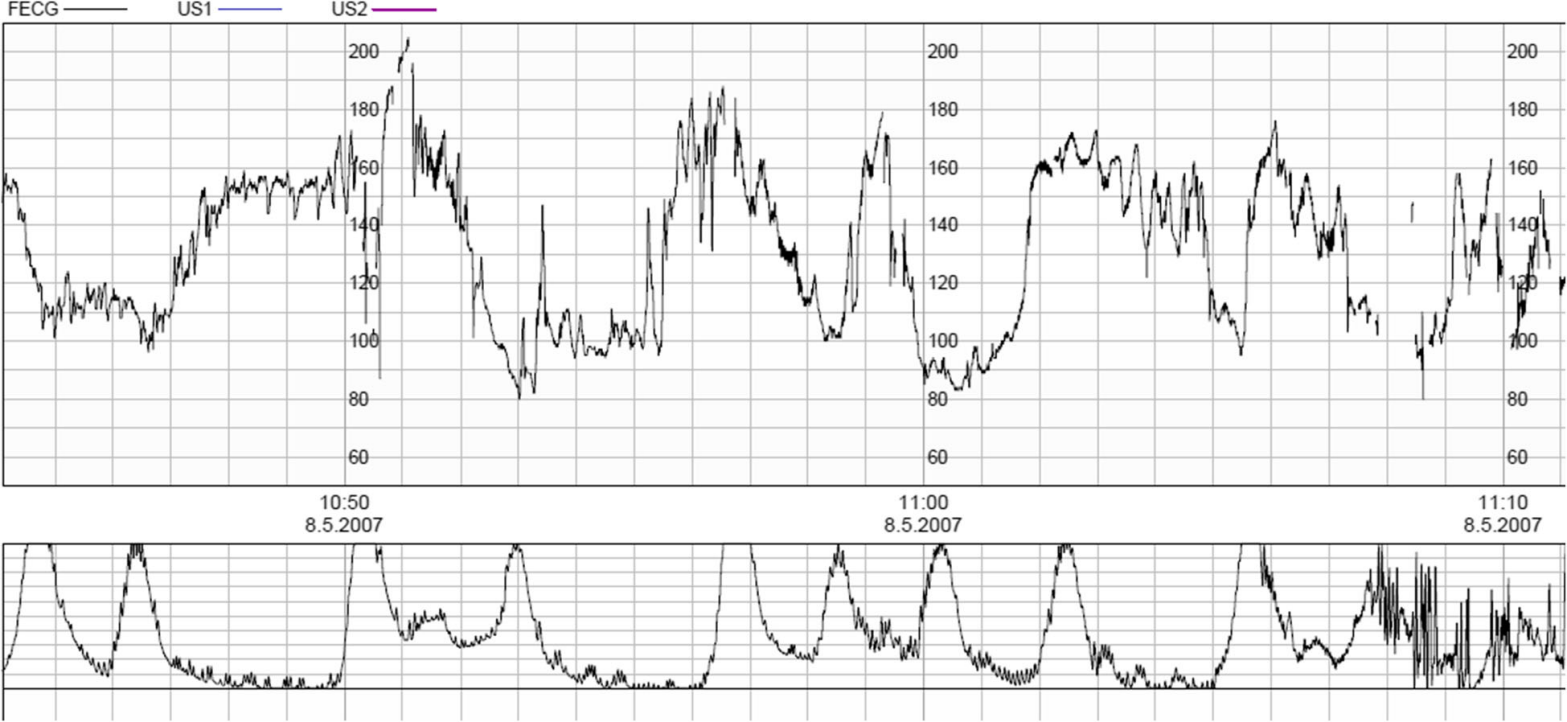
An example of complicated variable decelerations, their duration more than 60 s and depth more than 60 beats per minute

Two experienced obstetricians (OP and JU) evaluated the CTG traces, blinded to fetal presentation, actual mode of delivery and neonatal outcome. Only 60 min of tracing, immediately preceding either vaginal or emergency cesarean delivery, were evaluated, and thus the tracings were from the first and second stages of labor. As profound CTG changes are almost always observed immediately before birth, the decelerations of the final ten minutes of the tracing were not included in the classification unless the trace included a severe bradycardia before delivery. The obstetricians used the FIGO three-tier classification published in 1987 [7, 28] and provided their estimate on the baseline variability, presence of accelerations, late and complicated variable decelerations, and prolonged decelerations. The details of the classification of trace features can be found on Table 1 and an example of complicated variable decelerations in Fig. 1. The obstetricians also estimated the number of uterine contractions per 10 min. After independent evaluation of the traces, the obstetricians evaluated the traces together and formed a consensual interpretation, which was used in comparing the different features of the traces between breech and vertex deliveries. Third person (ET) collected the data from the mother’s medical records, which included summary information of the newborn. Pediatric records were also examined, if the child had health problems. During the study period, delayed cord clamping was not yet adapted in our hospital, and thus all the umbilical cord pH samples were taken after immediate cord clamping.

**Table 1:** Classification of the fetal heart rate tracings. Based on FIGO 1987 guidelines [7, 28]

Classification	Basal heart rate (bpm)	Baseline variability (bpm)	Decelerations
Normal	110–150	5–25 Accelerations	Early uniform decelerations Variable decelerations (duration <60 s and depth of <60 bpm)
Suspicious	100–110 150–170	<5 >25	Variable decelerations (duration <60 s and depth of <60 bpm)
Pathological	>170 <100 for >3 min	<5 for >60 min Sinusoidal pattern	Complicated variable decelerations (duration >60 s or depth >60 bpm)^a^ Late uniform decelerations

^a^See Fig. 1

All statistical analyses were performed using SPSS for Windows version 21.0 (IBM Corp., 2012. Armonk, NY, USA). Quantitative data were expressed as means or medians with minimum and maximum values. The results of categorical variables were described by percentages. The Student’s *t*-test and chi-square test were used as appropriate. Binary logistic regression analyses were performed to calculate odds ratios using forward logistic regression. A p-value of less than 0.05 was considered statistically significant. All p-values are two-tailed.

## Results

There were no differences between the groups in mothers’ mean age, duration of pregnancy, or in the prevalence of chronic illnesses or gestational complications, but significantly more mothers were primiparous in the breech group. Oxytocin augmentation was used more often in breech deliveries, but the difference was not statistically significant, and the presence of uterine tachysystole (defined as more than five contractions per 10 min) was similar in both groups. Unlike breech deliveries, vertex deliveries were assisted with vacuum extraction, and thus breech deliveries ended in an emergency CS significantly more often. Data concerning maternal background and labor are detailed in Table 2. The primary indication for intervention was most often suspected fetal asphyxia and did not differ between the groups (66.7 % of the interventions in the breech group and 70.8 % in the vertex group, *p* = 0.755).

**Table 2:** Demographic data and parameters concerning the mother and the delivery

	Breech deliveries *n* = 108		Vertex deliveries *n* = 108		*P* value
	Mean or n	% or range	Mean or n	% or range	
Maternal age (years)	28.9	18, 44	29.7	18, 46	0.224
Duration of pregnancy (weeks + days)	39 + 5	37 + 0, 41 + 6	40 + 1	37 + 0, 42 + 2	0.007
Primiparous	75	69.4	53	49.1	0.002
Chronic illness	12	11.1	11	10.2	0.825
Gestational diabetes	12	11.1	7	6.5	0.230
Pre-eclampsia	3	2.8	2	1.9	0.651
Spontaneus vaginal delivery	84	77.8	84	77.8	1
Operative delivery	24	22.2	24	22.2	1
- Vacuum extraction	None		13	12.0	<0.001
- Emergency CS	22	20.4	8	7.4	0.006
- Rush emergency CS	2	1.9	3	2.8	0.651
Induction of labor	15	13.9	23	21.3	0.153
Neuraxial analgesia	72	66.7	79	73.1	0.299
Oxytocin augmentation	82	75.9	70	64.8	0.074
Uterine tachysystole^a^	20	18.5	18	16.7	0.721

Legend: Comparison between breech and vertex intended vaginal deliveries in a Finnish tertiary hospital, 2007–2009

^a^Defined as more than five contractions per 10 min

Significantly more infants in the breech group had low Apgar score at the age of one minute, but at the age of five minutes there was no difference between the groups. Mean umbilical artery pH was lower in the breech group and pH 7.10 or lower was seen more often in the breech group. Conversely, no differences between the groups in admittance to neonatal intensive care unit, perinatal infections, or overall morbidity were observed. Details of the neonates are presented in Table 3.

**Table 3:** Neonatal outcomes

	Breech deliveries *n* = 108		Vertex deliveries *n* = 108		*P* value
	n or mean	% or range	n or mean	% or range	
1 min Apgar					<0.001
- Less than 4	11	10.2	3	2.8	
- 4–6	22	20.4	4	3.7	
- 7 or more	75	69.4	101	93.5	
5 min Apgar					0.408
- Less than 4	None		None		
- 4–6	4	3.7	2	1.9	
- 7 or more	104	96.3	106	98.1	
Umbilical cord pH	7.23	6.80, 7.46	7.27	7.05, 7.42	0.007
Cord pH ≤ 7.10	10	9.5	2	1.9	0.015
Cord pH ≤ 7.00	2	1.9	None		0.162
NICU admittance	4	3.7	4	3.7	1
Perinatal infection	10	9.3	6	5.6	0.299
Any illness or need for monitoring	24	22.2	16	14.8	0.161
Birthweight	3280	1935, 4350	3600	2320, 4670	<0.001

Legend: Comparison between breech and vertex intended vaginal deliveries in a Finnish tertiary hospital, 2007–2009

According to FIGO classification, traces from breech deliveries tended to display pathological patterns more often compared to vertex deliveries, but the difference was not statistically significant. However, breech delivery traces displayed decreased (less than 5 beats per minute) variability more often than traces from vertex deliveries, and late decelerations were more common in the breech group. There was no difference between the groups regarding frequency of complicated variable decelerations, prolonged decelerations or any abnormal decelerations (late, prolonged or complicated variable decelerations) grouped together. Details of the trace interpretations are presented in Table 4. Further analyses of infants displaying pathological trace patterns showed that 31 of the 45 breech infants and 19 of the 32 vertex infants were delivered spontaneously (*p* = .389). Infants that had displayed pathological trace showed more often one-minute Apgar score of six or less in the breech group (40 % of infants in the breech group and 18.8 % of the infants in the vertex group, *p* = .047), but there were no statistically significant differences between the groups regarding five-minute Apgar score, incidence of low cord pH or admission to neonatal intensive care unit.

**Table 4:** Expert interpretations of cardiotocography tracings

	Breech deliveries *n* = 108		Vertex deliveries *n* = 108		*P* value
	n	%	n	%	
FIGO classification					0.150
- Normal	29	26.9	39	36.1	
- Suspicious	34	31.5	37	34.3	
- Pathological	45	41.7	32	29.6	
Decreased variability	29	26.9	9	8.3	<0.001
Accelerations	84	77.8	94	87.0	0.074
Late decelerations	15	13.9	3	2.8	0.003
Complicated variable decelerations	58	53.7	62	57.4	0.584
Prolonged decelerations	26	24.1	29	26.9	0.639
Any abnormal decelerations^a^	69	63.9	66	61.1	0.673

Legend: Comparison between breech and vertex intended vaginal deliveries in a Finnish tertiary hospital, 2007–2009

^a^Late, complicated variable or prolonged decelerations or a combination of these

In order to study whether breech presentation, oxytocin use, pathological trace or any of the CTG features independently associate with adverse neonatal outcome in the study material, primary suboptimal neonatal outcome was defined as umbilical artery pH 7.10 or lower or five-minute Apgar score lower than 7. Using this definition, 13 infants in the breech group and 4 in the vertex group had suboptimal neonatal outcome (*p* = 0.023). Low five-minute Apgar score was associated with abnormal CTG trace; pathological in three out of four breech cases and one out of two vertex cases, and the rest two traces were defined as suspicious. Neonatal acidemia was likewise associated with pathological trace in the vertex group, as both two infants suffering from acidemia had displayed a pathological trace. However, in the breech group, acidotic cord pH was measured in seven infants with a pathological trace, two infants with a suspicious trace, and one infant with a normal trace. The infant displaying normal trace and acidotic pH was born to a healthy primiparous mother in spontaneous delivery on the 39th week of her pregnancy. Breech presentation was not diagnosed until labor, and the mother gave birth vaginally to a healthy baby weighing 3290 g, displaying cord pH 7.08, Apgar score of 7 at the age of one minute, and 9 at the age of five minutes. Further monitoring was not required.

Univariate analysis of the entire study population showed that suboptimal neonatal outcome was associated with pathological FIGO classification of CTG trace. Similarly, absence of accelerations, presence of late decelerations and presence of complicated variable decelerations as well as breech presentation were associated with suboptimal neonatal outcome. However, decreased trace variability, oxytocin augmentation, or uterine tachysystole were not significant predictors of adverse neonatal outcome. Odds ratios of univariate analyses are detailed in Table 5. Multivariable analysis revealed that complicated variable decelerations (OR 16.1, 95 % CI 2.1–124.8) and breech presentation (OR 4.1, 95 % CI 1.2–13.2) were independent risk factors for suboptimal neonatal outcome.

**Table 5:** Factors associated with primary neonatal outcome

	Primary neonatal outcome							
	Normal *n* = 199		Suboptimal^a^ *n* = 17		*p* value univariate analysis	OR univariate analysis	95 % CI univariate analysis	*p* value multivariable analysis
	n	%	n	%				
Pathological CTG trace in 3-tier FIGO classification	65	32.7	12	70.6	0.004	4.9	1.7–14.6	
Decreased variability	33	16.6	5	29.4	0.191	2.1	0.7–6.3	
Accelerations absent	31	15.6	7	41.2	0.012	3.8	1.3–10.7	
Late decelerations	14	7.0	4	23.5	0.027	4.1	1.2–14.1	
Complicated variable decelerations	104	52.3	16	94.1	0.010	14.6	1.9–112.3	0.008
Prolonged decelerations	49	24.6	6	35.3	0.337	1.7	0.6–4.8	
Vacuum extraction or emergency CS	43	21.6	5	29.4	0.460	1.5	0.5–4.5	
Primiparity	117	58.8	11	64.7	0.635	1.3	0.5–3.6	
Oxytocin augmentation	140	70.4	12	70.6	0.984	1.0	0.3–3.0	
Uterine tachysystole	35	17.6	3	17.6	0.995	1.0	0.3–3.7	
Breech presentation	95	47.7	13	76.5	0.031	3.6	1.1–11.3	0.020

Legend: Trace details and other obstetrical factors and their connection to primary neonatal outcome in breech and vertex intended vaginal deliveries in a Finnish tertiary hospital, 2007–2009

^a^Cord pH ≤7.10 or Apgar score at the age of five minutes <7

Immediate neonatal depression was seen in 40 infants, as 33 infants in the breech group and seven in the vertex group displayed Apgar score of less than seven at the age of one minute. Multivariable analysis showed that the factors independently associated with immediate neonatal depression were pathological trace (OR 3.2, 95 % CI: 1.5–6.7, *p* = 0.002) and breech presentation (OR 5.9, 95 % CI: 2.5–14.3, *p* < 0.001).

Uterine tachysystole was associated with pathological trace. 57.9 % of traces were deemed pathological when uterine tachysystole was found, whereas only 30.9 % of traces with normal uterine contractility were deemed pathological (*p* = 0.002). Conversely, oxytocin use did not seem to increase trace pathology, as pathological trace was seen in 38.8 % of oxytocin-augmented deliveries, compared to 28.1 % of deliveries with spontaneous labor (*p* = 0.134).

## Discussion

This cohort study of 216 deliveries showed that the CTG tracings from deliveries with the fetus in breech presentation displayed decreased variability and late decelerations more often than tracings from vertex deliveries. Moreover, a tendency to show pathological patterns (according to FIGO 1987 classification) more often was observed, although the difference was not statistically significant. In addition, breech presentation was seen to predispose infants to low Apgar score at the age of one minute, but there was no association with low Apgar score at the age of five minutes. Mean umbilical cord pH was lower in the breech group, but the incidence of severe acidemia (pH ≤7.00) did not differ between the groups.

Several ominous trace features were associated with neonatal depression. Especially pathological trace was shown to predict suboptimal Apgar score at the age of one minute, and complicated variable decelerations predicted neonatal depression (defined as umbilical artery pH 7.10 or lower or Apgar score less than seven at the age of five minutes). Breech presentation predisposed infants to both outcomes.

In this study, the effect of primiparous mothers having more compromised labor and deliveries should be reduced, as the mode of delivery was operative equally often between the groups. However, the weaknesses of this study include analyses not being adjusted by primiparity, which may cause bias in favor of the vertex group. Additionally, due to the study design, the interpreters did not know whether the tracings represented the second stage of labor or not, which may have affected the trace interpretations. Furthermore, the last hour of tracing before the delivery does not represent the entirety of labor, and at least in some cases longer tracings may have been more informative.

Although vaginal breech delivery has been consistently shown to predispose infants to low Apgar score at the age of one minute [14, 16, 29], the implication of this parameter is of minor importance. In order to enhance the clinical significance of the neonatal parameters, Apgar score at the age of five minutes and cord pH were chosen. However, this definition has similar problems, as the case of the breech infant with mildly acidotic pH and completely normal short-term outcome demonstrates. Furthermore, as our primary outcome variable included the cord pH, the results are biased in favor of the vertex group, as breech infants display lower cord pH values [14, 29]. Still, long-term data on infant health is not readily available, and likely as a consequence, primary neonatal outcome is most often used to study obstetrical management protocols. Infants in both groups were very healthy in general: outcomes that have been used to demonstrate neonatal morbidity such as severe birth trauma, Apgar score at the age of five minutes <4, or base deficit ≥15 [17], were not present in the study population.

The sample size of this study was mainly limited by the relatively low incidence of intended vaginal deliveries. The rate of intended vaginal deliveries in breech presentation has declined also in the study hospital [29] and thus is slightly lower than in other centers producing new research data on vaginal breech deliveries [27, 30]. Including additional centers would have increased the study population, but this would have reduced the objectivity and reproducibility of the trace interpretation, as it would have been impossible to form both independent and consensual evaluations of all the traces. In addition, including limited years and only one center, the management protocols of the deliveries were uniform and controlled.

We are aware that renewed FIGO guidelines have been introduced, but this study was conducted before those were published, which is why the 1987 guidelines were used. However, this study demonstrates the feasibility of the standardized guidelines in predicting neonatal depression also in vaginal breech delivery. Presumably the new classification will be even more reproducible and thus more effective and encourages further research. Furthermore, complicated variable decelerations are not recognized in the 2015 guidelines but instead categorized as late decelerations [31], which is appropriate also in light of this study, as they were identified as a risk factor for neonatal depression. On the other hand, the new guidelines have not yet been scientifically evaluated, and appropriate testing remains a challenge before adapting them into clinical setting.

Oxytocin use has been associated with adverse perinatal outcome in breech deliveries [32], and some institutions disfavor the use of oxytocin augmentation in breech deliveries, considering failure to progress in labor an indication for cesarean delivery [13]. In this study, neither oxytocin augmentation nor uterine tachysystole was associated with adverse neonatal outcome, which may be due to a low threshold to intervene with pathological CTG traces that in turn were associated with uterine tachysystole. However, vertex fetuses may have been, in some cases, quickly delivered by vacuum extraction when signs of fetal distress are observed in the second stage of labor, unlike breech fetuses, which were delivered by slower CS in similar circumstances. After the decision to deliver by an emergency CS is made, oxytocin infusion is discontinued and, in selected cases, tocolysis is administered, which may result in intrauterine recovery of the fetus from short-term asphyxia. This may cause the actual effect of oxytocin and uterine tachysystole on neonatal depression to be underestimated, especially in the breech group.

Although this study showed that breech presentation predisposed infants to low Apgar score at the age of one minute, another Finnish study demonstrated that vaginal breech delivery is associated with low Apgar score at the age of both one and five minutes, but the long-term health of these children is as good as that of children born vaginally in vertex presentation [33]. Some studies have demonstrated inferior immediate neonatal outcome in vaginal breech delivery [16, 17] and thus the frequent CTG pathologies may signal more frequent fetal distress. Even so, our results encourage attempting vaginal breech delivery in selected cases, as the neonatal outcome was comparable in both groups despite the higher incidence of ominous trace features in the breech group. Additionally, as pathological trace according to FIGO classification was shown to predict low Apgar score at the age of one minute and complicated variable decelerations alerted of prolonged fetal distress, continuous cardiotocography monitoring provides a tool for timely intervention in these cases. However, this is the first comparative study on cardiotocography in breech and vertex deliveries. More research is needed to form safe guidelines in managing vaginal breech delivery, should one attempt it. As the numbers of vaginal breech deliveries per institution are relatively small, multicenter studies or meta-analyses of uniform studies could provide the data needed.

## Conclusion

Cardiotocography tracings from breech deliveries display decreased baseline variability and late decelerations more often than tracings from vertex deliveries, but the association of these trace features with neonatal asphyxia is not clear. Trace pathology according to FIGO 1987 classification predicts neonatal depression and especially complicated variable decelerations seem to signal fetal distress, but also breech presentation seems to be an independent risk factor for neonatal depression.

## Abbreviations

BPM: Beats per minute
CI: Confidence interval
CS: Cesarean section
CTG: Cardiotocography
FIGO: International Federation of Obstetrics and Gynaecology
OR: Odds ratio
